# An Improved Sampling and Baiting Method for *Phytophthora tropicalis* and *P. heveae* Detection in *Macadamia integrifolia*

**DOI:** 10.3390/plants13192687

**Published:** 2024-09-25

**Authors:** Christopher M. Ference, Lisa M. Keith

**Affiliations:** 1 Daniel K. Inouye U.S. Pacific Basin Agricultural Research Center, Agricultural Research Service, United States Department of Agriculture, Hilo, HI 96720, USA; christopher.ference@usda.gov; 2 Oak Ridge Institute for Science and Education, 1299 Bethel Valley Road, Oak Ridge, TN 37830, USA

**Keywords:** macadamia quick decline, baiting, oomycete, tree sampling, method development

## Abstract

Macadamia nuts are, economically, the second most important crop in the state of Hawai’i. A recent decline in yield and acreage has been attributed to insect damage and diseases such as Macadamia Quick Decline (MQD) caused by *Phytophthora tropicalis* and *P. heveae*. To develop an improved methodology for the diagnosis and treatment of MQD, investigations were undertaken to better understand the pathosystem of the disease. These investigations included sampling from multiple locations from sectioned trees utilizing two methods of tissue collection and isolations using two baiting techniques. The collection of tissue from the cambium and phloem of trees after scraping away the bark and in locations of recent or current sap exudation using a narrow diameter steel awl proved to be an efficient means for the molecular detection of the MQD pathogens from infected trees exhibiting MQD symptoms. In addition, a more efficient and cost-effective baiting method using apple puree was developed.

## 1. Introduction

The entire U.S. domestic production of macadamia nuts occurs in Hawai’i and macadamia (*Macadamia integrifolia* Maiden & Betche) is the second most economically important crop in the state, with a total value of USD 33.2 million in 2022 [1]. This was, however, a significant drop from the previous year’s value of USD 65 million. While a portion of this value reduction can be attributed to a drop in the unit price of macadamia nuts, the loss of acreage and decline in yield are also significant factors. Most of the state of Hawai’i’s 16,200 cultivated acres of macadamia are on the island of Hawai’i, but this is a decline from a historic high of 17,000 acres in 2021 [1]. Similarly, 2022 saw a utilized production of 18,850 tons, which was down from 26,450 tons in 2021, indicating a drop from 1.56 to 1.16 tons of utilized production per acre [1]. This decline in yield and acreage has been attributed to insect and other animal damage as well as to disease [1].

**Figure 1:**
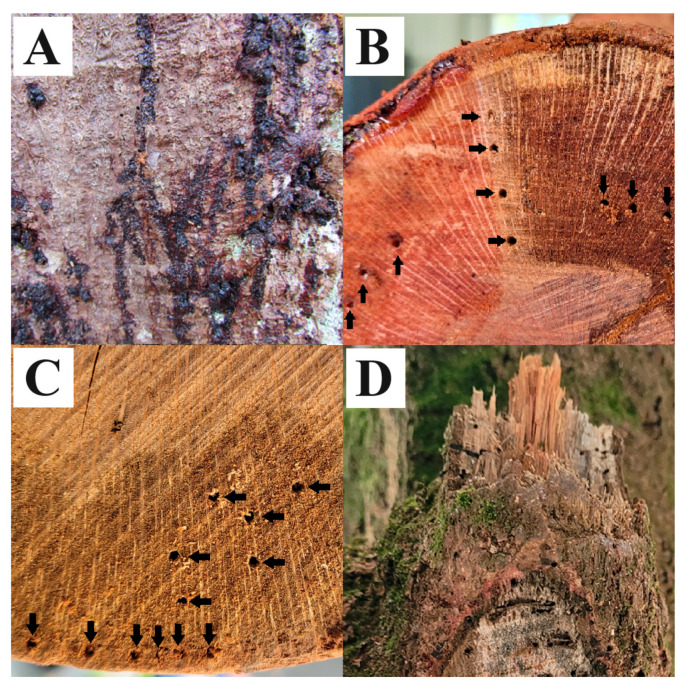
(**A**) MQD-symptomatic ‘bleeding’ = dried exuded sap on main trunk; (**B**) drill sampling from wood; up-pointing arrows indicate drill holes from healthy looking sapwood, right-pointing arrows indicate drill holes from boundary sapwood, and down-pointing arrows indicate drill holes from MQD-symptomatic dried stained sapwood; (**C**) shallow and deep sampling; down-pointing arrows indicate drill holes from vascular cambium and phloem, and left-pointing arrows indicate drill holes from stained sapwood; (**D**) unhealed branch wound with insect bore holes.

One of the most impactful diseases in terms of tree health and yield is Macadamia Quick Decline (MQD). MQD is a disease caused by the oomycete plant pathogen *Phytophthora* (species *tropicalis* and *heveae*), which is widespread on the island of Hawai’i, having been detected infecting macadamia nut trees in all significant areas of commercial cultivation. Symptoms and signs of MQD include sap exudate (bleeding) (Figure 1A), wood staining (Figure 1B,C), and chlorosis, followed by the partial or complete browning of the crown, which occurs suddenly and typically eventuates in tree death within months [2]. MQD was first described in Hawai’i in 1991 [3], with *Phytophthora tropicalis* not identified as the causative agent until 2010 [4]. Following a new outbreak of MQD-like symptoms in macadamia fields on Hawai’i Island, infection by a second species of *Phytophthora*, *P. heveae*, was also determined to result in MQD [5]. While not significant with respect to macadamia cultivation in Hawai’i, *Phytophthora cinnamomi* is another important oomycete causing disease on macadamia nut trees. Found widely in Africa and Australia, killing or severely damaging thousands of trees, a *Phytophthora cinnamomi* infection of the tree trunk results in cankers, necrotic lesions, and gummosis [6,7,8].

Currently, there exists little in the way of advisable management strategies for the mitigation of the effects of MQD besides non-curative treatments with phosphorous acid and the physical removal of infected trees. Rapid and accurate diagnosis is the first critical step needed for successful disease management in the field. Timely response can prevent the spread of disease. To develop an improved methodology for tree diagnostics, investigations were undertaken to better understand the pathology of MQD and the colonization patterns of *P. tropicalis* and *P. heveae* in macadamia, which included MQD-positive tree sectioning and sampling.

## 2. Results

### 2.1. Tree Sampling Method for qPCR Detection of Phytophthora

**Figure 2:**
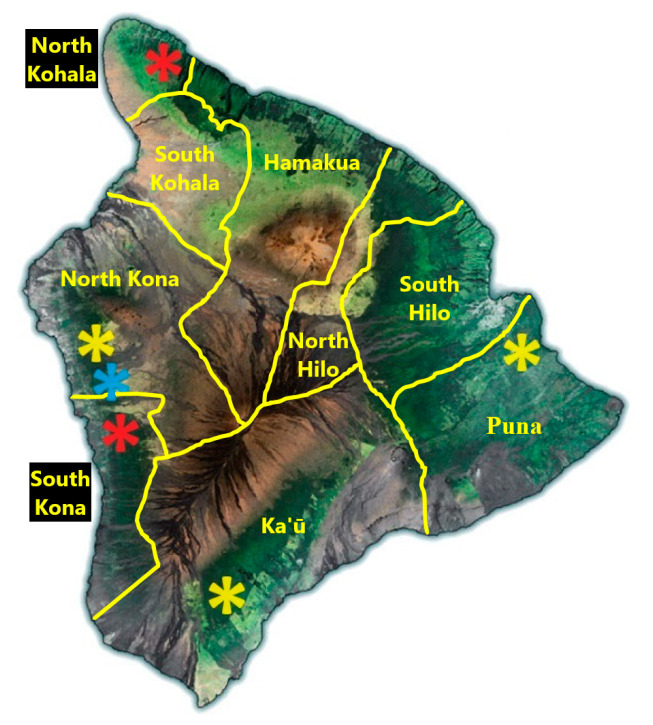
Locations on the island of Hawai’i of sampled trees exhibiting MQD symptoms. Blue asterisks = *P. tropicalis*. Red asterisks = *P. heveae*. Yellow asterisks = both species of *Phytophthora*.

**Figure 3:**
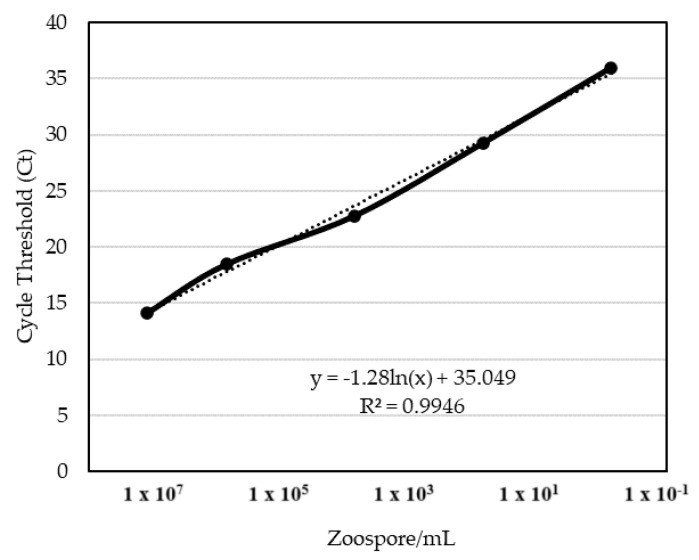
Comparing zoospore concentrations of *Phytophthora heveae* with their cycle threshold (Ct) following qPCR. The formula for the best-fit slope is y = (−1.28 × ln(x)) + 35.049 which is represented by the dotted black line, with an R^2^ confidence value of 0.9946, where 1 = perfect confidence.

To determine the presence of *Phytophthora*, tissue samples collected from macadamia trees were processed and analyzed using qPCR. Results for the positive detection of *Phytophthora* are reported by the strength of their cycle threshold (Ct) value. The Ct value represents the fractional number of iterative cycles of target nucleic acid duplication required for a fluorescent signal to exceed a background level threshold and is therefore inversely proportional to the amount of target nucleic acid initially in the sample. Data are reported as the Ct value when sample amplification passed the background level threshold. From 39 trees sampled from 4 districts of the Island, 22 were found to be positive for *P. tropicalis* (8), *P. heveae* (13), or both (1) (Figure 2 and Table 1). From these 22 *Phytophthora*-positive trees, a combined total of 162 samples positive for *Phytophthora* were obtained. Using Method 1, 7 of 20 samples returned positive qPCR results (Ct ≤ 35) for the detection of *P. heveae*, while 14 samples collected using Method 2 returned positive results. All 7 matched pairs which had a positive result using Method 1 also had a positive result when using Method 2, while 7 other matched pairs only had a positive result when using Method 2. Six matched pairs were negative using both Method 1 and Method 2. The qPCR Ct values of the 7 matched pairs where both Method 1 and Method 2 returned a positive detection of *P. heveae* are shown in Table 2. Lower Ct values were consistently obtained from tissue samples collected using Method 2. The Ct values from tissue samples collected using Method 2 were 3.71 ± 0.69 lower than those from Method 1 samples when both samples returned a positive result, indicating 79–99% more target DNA collected using Method 2, with Ct values correlating to differences in the amount of nuclear material collected equivalent to concentrations of between 1.164 × 10^4^ (matched pair #4) and 1.154 × 10^5^ (matched pair #1) *P. heveae* zoospores per milliliter (Table 2 and calculations using the best-fit equation found in Figure 3).

Two samples from a tree in North Kohala that was exhibiting fresh bleeding from a main branch were taken rootward from the source of exudate and resulted in Ct values of 21 and 20 for *Phytophthora* (equivalent to 1.15 × 10^5^ and 5.28 × 10^4^ zoospores/mL, respectively, using the equation found in Figure 3). A macadamia tree in Hōlualoa (North Kona district) exhibiting crown decline and bleeding was sampled rootward of a bleeding site on a branch, and this returned a positive cycle threshold of 18 (6.09 × 10^5^ zoospores/mL) for *Phytophthora*, later identified as *P. tropicalis*. Two trees in Nā’ālehu (Ka’ū district) exhibiting bleeding from the main trunk were sampled, both rootward from the source of exudate, and returned positive cycle thresholds of 20 and 22 (5.28 × 10^4^ and 2.68 × 10^4^ zoospores/mL, respectively) for *Phytophthora*, later identified as *P. heveae* and *P. tropicalis*, respectively.

### 2.2. Baiting Method for Retrieval of Viable Phytophthora

Of the 30 *P. tropicalis*-infected tree samples which were baited using the whole apple method, viable *Phytophthora* growth was isolated from 4 (13.3%). For the 30 *P. heave* samples, only 3 samples (10.0%) were successfully baited using the whole apple method. Using the apple puree method, an increase of two to three times more successful baits was achieved, with 10 *P. tropicalis* samples (50.0%) and 9 *P. heveae* samples (45.0%) viable. When *Phytophthora* was successfully baited out from samples, growth on plates using apple puree emerged within 2 to 3 days. In contrast, symptoms on apples typically took 3 to 5 days to present, followed by 1 to 2 days before *Phytophthora* growth on the isolation plate containing the excised piece of apple tissue could be observed. While apples could be halved, cutting a hole and including more than one tree tissue sample per apple half risked having all samples lost should one of the holes become contaminated with a fast-growing non-*Phytophthora* organism. This risk was eliminated by using apple puree in tubes for each individual tissue sample. The sequencing of DNA isolated from pure cultures of suspected *Phytophthora* species recovered from macadamia nut tree tissue samples baited with apple puree returned positive results for both *P. heveae* and *P. tropicalis*. *Phytophthora heveae* CF060 was isolated from a commercial macadamia nut tree in Hala’ula, North Kohala, Hawai’i, exhibiting bleeding and partial crown decline symptoms consistent with MQD. *Phytophthora tropicalis* CF667 was isolated from a commercial macadamia nut tree in Hōlualoa, North Kona, Hawai’i, that was also exhibiting typical MQD symptoms, including total crown decline. Both trees showed mechanical wounding of the main trunk due to weed removal equipment with the Kohala tree, and excessive pruning with the Hōlualoa tree.

## 3. Discussion

As there are no MQD-resistant cultivars of macadamia currently being commercially grown in Hawai’i and fungicide applications have demonstrated only the ability to delay inevitable tree death, management recommendations include the removal of diseased trees and cultivation practices which minimize wounding [9,10,11]. Foliar and drench applications of phosphorous acid have therapeutic effects on macadamia trees infected with *P. tropicalis* [9] and are also effective against additional species of *Phytophthora* infecting other plants [12]. Management methods using an irrigation delivery of fungicides to combat *Phytophthora* species are generally not applicable in Hawai’i because macadamia trees are not typically irrigated. To optimize the effectiveness of management strategies, the early and accurate detection of MQD-causing *Phytophthora* is essential.

Sampling from various parts of macadamia nut trees suspected to be infected by *Phytophthora* indicated multiple trends for how a positive qPCR result for *Phytophthora* detection could best be achieved. In general, sampling from the cambium and living phloem of a tree produced stronger (Ct 3.71 ± 0.69 lower) positive results than sampling from deeper into the wood of the tree at the same location using a drill and sterilized drill bit. While it was possible, though infrequent, to obtain a positive detection result from a sample composed solely of wood tissue, bypassing the bark and vascular cambium by sampling directly from the face of a lateral cut across the trunk, positives tended to be weak, and results were often negative. This suggests that though the molecular detection of MQD-causing *Phytophthora* species can be obtained with drill sampling, it is the shallower tissues, the phloem and the cork and vascular cambium, which are most strongly contributing to the positive result. By implication, this further suggests that while *Phytophthora* infiltrates sapwood over time, collecting samples from the shallower living tissues of the tree serves as a much better option for rapid and accurate detection results. Samples from which viable *Phytophthora* were retrieved were also samples which returned the lowest qPCR Ct values for the positive detection of *Phytophthora*, with results typically being in the high teens to low twenties.

Though bleeding can occur after mechanical damage or due to other types of stress, when *Phytophthora* was detected in a tree, samples taken from a proximity to sources of ongoing or recent bleeding typically produced the strongest positive results for *Phytophthora* detection from that tree. Tissue samples collected rootward from the proximity of sap leakage from trees in North Kohala district, Nā’ālehu (Ka’ū district), and Hōlualoa (North Kona district) returned the strongest positive cycle thresholds of all samples collected from those trees. From the *P. heveae*-positive tree, a total of 74 samples were taken from various parts of the tree during a complete dissection, and none produced a stronger Ct result than the shallow knife/awl-collected sample (Method 2) taken immediately rootward from the site of recent bleeding. From the *P. tropicalis* tree, 81 total samples were taken during a tree dissection, with only 3 producing a stronger Ct result, 2 of which were taken from the same height as the site of bleeding, though at different points around the circumference of the trunk. The third strongest Ct result was obtained from a lower section of the trunk in an area with several insect bore holes (Figure 1D).

A third trend observed were positive detections of *Phytophthora* with high Ct results around sites of mechanical wounding. While not a symptom of MQD, wounds could conceivably serve as an infection court for the development of disease due to *P. tropicalis* and *P. heveae*. For the Nā’ālehu tree which was found to be infected by *P. heveae*, samples taken from around the jagged and insect bore hole perforated wound from the collar of a broken branch (Figure 1D) returned numerous positive detections for *Phytophthora* with Ct values in the mid- to low twenties. Increasing Ct values, indicating a decrease in pathogen concentration, correlated with increasing distance from the wound. For trees surveyed from six sites around Hawai’i Island (Figure 2), wounds, due to animals such as feral pigs, strong winds, heavy equipment for weed control or nut harvesting, or excessive pruning without afterward using a wound sealant, were often found on trees which were later determined to be infected with one of the two species of *Phytophthora* responsible for causing MQD. While some of these phenomena have been observed in other pathosystems in Hawai’i [13], additional surveying and sampling are required to firmly establish a link between wounds and the development of MQD.

Using sterile apple puree to bait out viable *Phytophthora* from infected macadamia nut tree tissue was successful using the newly developed method. Experimental investigations of baiting samples taken from the same location as previously successfully baited samples had a 47.5% success rate (19 out of 40) of viable *Phytophthora* retrieval, which was considerably better than the whole apple method success rate (7 out of 60). The sequencing of pure cultures of *Phytophthora* isolated from MQD-infected trees and baited out using the apple puree method identified *P. tropicalis* and *P. heveae*, proving that both species of MQD-causing *Phytophthora* are capable of being baited out using this technique. When compared to the whole apple method for baiting out *Phytophthora*, apple puree is a method that is less costly, laborious, complex, and prone to contamination, produces faster results (2–3 days versus 4–7 days), and is more suited to the processing of many samples. The apple puree method is also conducive to field application, where samples from suspected *Phytophthora*-infected trees can be put immediately into pre-made vials containing sterile apple puree. The addition of antibiotics to the puree could be used to further protect against contamination; however, the preliminary testing of the addition of antimicrobial compounds (ampicillin, nystatin, and pentachloronitrobenzene [PCNB]) resulted in a reduced success rate of baiting using this method. More investigation is required to determine if additional fungicides could be added to improve this newly developed method.

## 4. Materials and Methods

### 4.1. Tree Sampling for Detection of Phytophthora

Multiple tissue samples from trees growing in several locations on the island of Hawai’i exhibiting MQD symptoms were collected (Table 1). Samples were collected from multiple locations on individual *Phytophthora*-positive trees, including at various heights from the ground, around the circumference of the trunk, from branches, at various depths, near wounds, in and around areas of stained wood (Figure 1B,C), and near sites of sap exudate. From a particular tree from the North Kohala district previously found to be infected with *P*. *heveae*, a total of 40 tissue samples were collected in two ways, with 20 samples collected using one of the two following methods. The first method involved a handheld power drill with a flame sterilized 1/8th inch (3.175 mm) diameter drill bit used to create a hole approximately 2–3 cm deep toward the center of the tree. Wood shavings from the bit were collected in a sterile 1.5 mL microcentrifuge tube. The second method involved the subsequent collection of tissue from the same location as the drill bore hole using an ethanol-sterilized knife to scrape away and discard an approximately 1 cm^2^ section of the rhytidome and an ethanol-sterilized scratch awl (5.6 mm diameter; Model A1, Malco Products, Annandale, MN, USA). The tissue, which included mostly periderm and secondary phloem, was collected in a sterile 1.5 mL microcentrifuge tube.

### 4.2. Real-Time PCR and Sequence Identification

Samples collected from the tissue of macadamia nut trees exhibiting symptoms of MQD using the two previously described methods were processed identically using the NucleoSpin Plant II kit and instructions (Macherey-Nagel; Düren, Germany). Purified DNA was evaluated using a TaqMan qPCR assay for the genus-level detection of *Phytophthora* [14]. A dilution series containing known concentrations of *Phytophthora* zoospores were processed using qPCR to establish a standard for correlating Ct values with pathogen nuclear material concentration. Species-level and morphological identification was confirmed by the molecular analysis of the 5.8S subunit and flanking internal transcribed spacers (ITS1 and ITS2) of rDNA amplified from DNA extracted from single-hyphal tip cultures with the ITS1/ITS4 primers [15], sequenced by Eurofins Genomics LLC (Louisville, KY, USA) and compared in GenBank.

### 4.3. Recovery of Viable Phytophthora

Over the course of the study, attempts to recover viable *Phytophthora* were made from 20 trees. Two methods were used to bait viable *Phytophthora* from macadamia tree tissue samples, including a traditional method of using whole apples and a novel method using sterilized apple puree. To assess the efficacy of the novel method to retrieve viable *Phytophthora* from plant tissue relative to the traditional method, tissue samples were collected from specific areas of trees where viable *Phytophthora* had been previously recovered using the traditional whole apple method. Locations for sample collection were near areas of fresh sap exudate and around wounds where previous samples had returned strong Ct values from qPCR (Figure 1A,D). From two representative trees from the Ka’ū district of the island of Hawai’i, a total of 100 baiting attempts were made, 50 from a tree infected with *P. tropicalis*, and 50 from a tree infected with *P. heveae*. An approximately 1 cm^2^ section of the rhytidome of an MQD-symptomatic macadamia tree was scrapped away with an ethanol-sterilized flat bladed knife. An ethanol-sterilized scratch awl was used to remove approximately 1.5 mL of tissue, including mostly periderm and secondary phloem, but with some secondary xylem, into a sterile 2 mL centrifuge vial. The 100 tissue samples were baited using two methods, with 60 samples (30 each from the *Phytophthora tropicalis*- and *heveae*-infected trees) processed using Method 1 (whole apple), and 40 samples (20 each from the *Phytophthora tropicalis*- and *heveae*-infected trees) processed using Method 2 (apple puree).

The first method used a modification of the traditional whole apple (*Malus domestica*) technique to retrieve viable *Phytophthora* from plant tissue [16]. Tissue samples were packed into the flesh of a green apple (*Malus domestica* × *M*. *sylvestris*) in a manner similar to previously described soil baiting techniques for *Phytophthora* [17]. Briefly, apples were surface-disinfested with 70% ethanol and halved, a cone shaped hole was cut into the peel surface of an apple half, plant tissue was placed inside the hole, the cone was returned, a sterile cotton ball saturated with sterile water was placed on top to prevent the plant tissue from drying out, and the entire construct was wrapped in parafilm to hold everything in place. Apple halves were placed in sealable plastic bags and the closed bags were placed in closed lidded plastic containers. The containers were left on the lab bench at room temperature for 4–6 days, until firm mottled browning symptoms developed on the peel near the cone incision. *Phytophthora* was isolated by excising a small amount of apple tissue from the advancing edge of the symptomatic browning and plating this tissue on 10% V8 agar which was then incubated at 25 °C under constant light. Hyphal tips of growth resembling the colony morphology of *Phytophthora* were transferred to antibiotic-amended water agar plates (200 mg L^−1^ ampicillin, 100 mg L^−1^ nystatin, and 20 mg L^−1^ pentachloronitrobenzene [PCNB]) and incubated for 1–2 days at 25 °C under constant light, and once confirmed pure, single hyphal tips were transferred to 10% V8 agar plates and incubated at 25 °C under constant light.

The second method involved injecting a room-temperature equilibrated 1 mL aliquot of sterilized apple puree into a microcentrifuge tube containing excised tree tissue samples. Apple puree was made from peeling, coring, and dicing a 70% ethanol surface-disinfested green apple (*Malus domestica* × *M*. *sylvestris*), followed by autoclaving for 25 min at 121 °C. Apple puree was stored in a darkened refrigerator (4 °C) if not immediately used. Vials containing tree tissue samples and apple puree were incubated for 2–3 days at 25 °C with constant light. After 2–3 days, using a sterile loop, apple puree was plated onto PCNB antibiotic-amended water agar plates, prepared as in the whole apple method. These plates were incubated for 2–3 days at 25 °C with constant light.

After 48 to 72 h, the antibiotic-amended water agar plates were examined under a dissecting microscope for mycelial growth characteristic of *Phytophthora*. Single hyphal tips of growth morphologically appearing to be either *P*. *heveae* or *P*. *tropicalis* were removed with a sterile scalpel and transferred to potato dextrose agar (PDA) plates, that were then placed in an incubator set at 25 °C and with constant light. Mycelial growth was collected via scraping with a sterilized metal loop from these incubated PDA plates after 24 to 48 h of growth and placed in 2 mL sample tubes containing six 2 mm diameter sterile plastic beads. Mycelia were macerated in a bead mill, and DNA was extracted from the macerate using a NucleoSpin Plant II kit (Macherey-Nagel, Düren, Germany) following the manufacturer’s protocols, followed by *Phytophthora* spp. detection using qPCR as previously described in Section 4.2. Samples producing positive results were sent for species identification, also as described in Section 4.2.

### 4.4. Statistical Analysis

Data were organized and graphed using Excel (Version 2407, Microsoft Corp., Seattle, WA, USA) and analyzed by the one-way analysis of variance (ANOVA) method using JMP statistical analysis software (version 16; SAS Institute, Cary, NC, USA). Matched-pair comparison method was applied for PCR cycle threshold comparison [18]. Differences among multiple groups were analyzed using Tukey’s HSD test. *p* < 0.05 was defined as statistically significant differences. All experiments were performed at least three times. Results are represented as mean ± standard error.

## 5. Conclusions

An economical, field-friendly detection and baiting method was developed for the rapid and accurate identification of *P. tropicalis* and *P. heveae* from macadamia. The shallow scraping of living tree tissue and the incubation of that tissue in apple puree constitute an efficient and more sensitive method for the high-throughput detection and baiting of MQD-causing *Phytophthora* species in macadamia nut trees, which allows for appropriate management actions to be carried out in a timely manner.

## Figures and Tables

**Table 1:** Sampling data for MQD-symptomatic (bleeding/decline) macadamia nut trees from the island of Hawai’i.

	Collection Location (Districts of Hawai’i Island)				
	Puna	North Kohala	Ka’ū	South Kona	North Kona
Total # trees sampled	3	2	13	4	17
# of trees positive for *P. heveae*	2	2	1	4	5
# of trees baited for viability	1	2	1	4	3
# of trees positive for *P. tropicalis*	1		1		7
# of trees baited for viability	1		1		6
Total # of *Phytophthora*-positive samples	3	40	15	5	99

**Table 2:** Selected matched-pair comparison of positive qPCR cycle threshold (Ct) values from two collection methods. All samples using Method 1 were collected from the main trunk, laterally through the bark and approximately 2–3 cm into the sapwood of the tree. All samples using Method 2 were collected from the same location where samples using Method 1 were first collected.

Method	Matched Pair						
	1	2	3	4	5	6	7
1 (drill)	23	24	25	32	24	28	33
2 (knife/awl)	20	21	23	26	22	25	26

## Data Availability

The original contributions presented in this study are included in the article. Further inquiries can be directed to the corresponding author.
